# Tuning the Effect of Chitosan on the Electrochemical Responsiveness of Lignin Nanoparticles

**DOI:** 10.1021/acsbiomaterials.2c01494

**Published:** 2023-05-16

**Authors:** Valeria Gigli, Eliana Capecchi, Cristina Tortolini, Andrea Isidori, Riccarda Antiochia, Raffaele Saladino

**Affiliations:** † Department of Experimental Medicine, Sapienza University of Rome, Viale Regina Elena 324, 00166 Rome, Italy; ‡ Department of Biological and Ecological Sciences, University of Tuscia, 01100 Viterbo, Italy; § Department of Chemistry and Drug Technologies, Sapienza University of Rome, Piazzale Aldo Moro 5, 00185 Rome, Italy

**Keywords:** lignin nanoparticles, chitosan, electrochemical nanodevices, sustainable electrochemistry

## Abstract

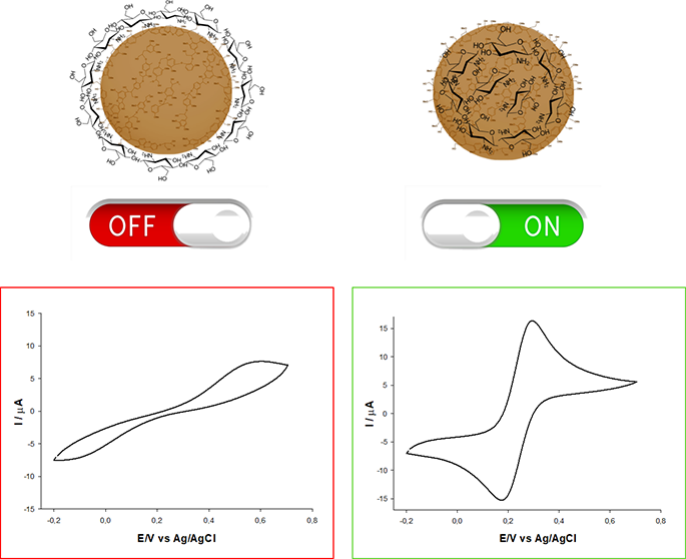

Chitosan and lignin mixed nanoparticles were prepared by layer-by-layer and nanoprecipitation methodologies as responsive platforms for sustainable biosensors. The novel nanoparticles showed effective chemophysical and electrochemical properties dependent on the preparation methodology, molecular weight of chitosan, and type of lignin. HOMO–LUMO energy gap calculations suggested the presence of structure–activity relationships between the electrochemical responsiveness and the order and orientation of lignin aromatic subunits and chitosan chains in the nanodevices.

## Introduction

Lignin is the most abundant polyphenol in nature. It is characterized by an amorphous form and complex chemical structure comprising phenylpropanoid units in a disordered sequence.^1^ The elemental composition of lignin is variable depending on the origin and isolation technologies.^2^ Pulp, paper, and biofuel industries generate lignin as waste in high amounts reaching around 50 million metric tons per year.^3,4^ However, 95% of the lignin is still treated as waste.^5^ Examples of the application of lignin in the design of electrochemical devices are reported.^6^^7^ Alkali lignin, kraft lignin, crosslinked cationic lignin, and lignosulfonates are used as conductive binders in lithium-ion batteries, gel polyelectrolytes, aqueous flow batteries, and energy storage and wearable electronic devices.^8−17^ In addition, the application of organosolv lignin as an immunosensor in electrochemical impedance spectroscopy and electrocatalysis is reported.^18−20^ The large-scale application of lignin is limited by the variable composition of the original material.^21^ This limitation is overcome by expensive pyrolytic techniques associated with high-temperature treatments (500–1300 °C).^22−28^ The self-assembly of lignin into ordered nanoparticles (LNPs) can be a promising alternative to traditional approaches due to the emergence of beneficial chemophysical, rheological, and electrochemical properties as a consequence of the supramolecular organization of the aromatic subunits of the polymer.^29^ It includes π–π HOMO–LUMO interactions with the formation of head-to-tail (J-type) and tail-to-tail (H-type) aggregates and the emergence of unprecedented electron-transfer behaviors.^30−32^ Currently, the applications of LNPs in biosensing are still limited to phototriggered and photoluminescence devices based on the immobilization of redox enzymes.^33−37^ In the latter case, the boosting effect associated with long-range electron-transfer (pseudo-DET) and mediated electron-transfer (MET) processes were reported and discussed in detail, focusing on the role played by low-molecular-weight mediators as diffusible shuttles from the enzyme to the bulk of the solution.^32,38,39^ In principle, the HOMO–LUMO energy gap in LNPs may be controlled by the selection of the starting lignin as well as by the presence of other polymers, such as polysaccharides.^30,40,41^ Polysaccharides act as spacers and structural orienting motifs in lignin aggregation, their effect being dependent on the deacetylation degree and molecular weight of the molecule.^30^ We recently reported that the HOMO–LUMO energy gap in lignin–chitooligosaccaride nanoparticles is modulated by the saccharide component.^30^ To the best of our knowledge, no data are available on the electrochemical behavior of lignin–chitosan nanoparticles (LNPs/CS). Here, we describe the preparation of different types of LNPs/CS by two alternative technologies, namely, layer-by-layer assembly and nanoprecipitation procedure, and their full characterization concerning cyclic voltammetry and impedance properties, with particular focus on structure (composition)/activity relationships. Three different technical lignins, kraft lignin (KL), organosolv lignin (OL), and enzymatic hydrolysis lignin (EHL), and chitosan samples with different molecular weights (CS), were used in the preparation procedure. The optimal electrochemical responsiveness was obtained by the nanoprecipitation technique using chitosan with a molecular weight of 50 kDa and kraft lignin. In the latter case, a reversible potential difference (Δ*E*) as high as 113 mV was obtained. These results open a new pathway for the use of lignin in the design of sustainable and low-cost electrochemical devices capable of selective recognition processes.

## Materials and Methods

### Materials

Organosolv lignin (OL), enzymatic hydrolysis lignin (EHL), and kraft lignin (KL) were obtained from Chemical Point. Chitosan (75% deacetylation degree) with different molecular weights of 5 kDa (CS1), 50 kDa (CS2), and 100 kDa (CS3) were purchased from Heppe Medical Chitosan GmbH. Reagents, including tetrahydrofuran (THF) and ethanol, were purchased from Sigma Chemical Co. (St Louis, MO) and used without further purification. Spectrophotometric analysis was performed with a Cary 60 UV–vis spectrophotometer equipped with a single-cell peltier thermostated cell holder, Agilent (Santa Clara). Data were elaborated with Cary Win UV software. Electrochemical measurements were performed in a conventional three-electrode thermostated glass cell (model 6.1415.150, Metrohm, Herisau, Switzerland) using a glassy carbon electrode (GCE, 6.1204.600 GC, diameter 2 ± 0,1 mm, Metrohm, Italy).

### Preparation of Lignin/Chitosan Nanoparticles by Nanoprecipitation Technology (Np-LNPs/CS)

Np-LNPs/CS were prepared by a slightly modified nanoprecipitation procedure using deionized water or acetic acid (1.0% water solution) as the antisolvent and THF/EtOH/H_2_O as the primary solvent.^30,42,43^ As a general procedure, lignin (1.0 g) in THF/EtOH/H_2_O (14.5 mL) was rapidly added to CS1 or CS2 (0.1 g) in deionized water (72.5 mL), or alternatively to CS3 (0.1 g) in acetic acid (1.0% water solution), under gentle mechanical stirring at 25 °C. Np-LNPs/CS were isolated after evaporation of the organic solvent under reduced pressure, followed by centrifugation (12,100 rpm for 10 min; 2 times) and freeze-drying for 24 h.

### Preparation of Lignin/Chitosan Nanoparticles by Layer-by-Layer Technology (LbL-LNPs/CS)

**LbL-LNPs/CS** were prepared by coating preformed LNPs (1.0 g) with CS1 or CS2 (0.1 g) in deionized water (72.5 mL), or alternatively with CS3 (0.1 g) in acetic acid (1.0% water solution) (72.5 mL), under orbital shaking at 25 °C for 24 h. **LbL-LNPs/CS** were isolated after centrifugation (12,100 rpm for 10 min 2×) and freeze-dried for 24 h.^31^

### UV–vis Absorption Spectra

The UV–vis absorption spectra of **Np-LNPs/CS** and **LbL-LNPs/CS** were measured with a Cary UV 60 spectrophotometer using the standard concentration of nanoparticles (0.1 mg/mL) in deionized water (3.0 mL) and in the working range of 190–600 nm at 25 °C under gentle stirring.

### Field-Emission Scanning Electron Microscopy (FE-SEM)

Field-emission scanning electron microscopy (FE-SEM) of **LNPs/CS** was performed by a ZEISS GeminiSEM500 at 5 kV. The sample (20 μL in deionized water) was dropped on specimen stubs, air-dried, and coated with gold by sputtering with an AGAR Auto Sputter Coater. Before the measurement, the sample was deposited with a chromium thin film (5 nm) by sputter-coating using a QUORUM Q 150T ES plus coater.

### Dynamic Light Scattering (DLS) and ζ Potential Analysis

The hydrodynamic diameter and ζ-potential were measured with DLS by suspending a freshly prepared sample in H_2_O using a Zetasizer Nano ZS (Malvern Instruments, Malvern, U.K.) apparatus equipped with a He–Ne laser (633 nm; fixed scattering angle 173°; 25 °C). Measurements were performed in triplicate at 25 °C.

### Electrochemical Characterizations

Electrochemical measurements were performed in a 10 mL conventional three-electrode thermostated glass cell (model 6.1415.150, Metrohm, Herisau, Switzerland) using a glassy carbon electrode (GCE) as a working electrode, an external Ag/AgCl/ KCl_sat_ electrode (198 mV vs NHE) as a reference electrode (cat. 6.0726.100, Metrohm, Herisau, Switzerland), and a glassy carbon rod as a counter electrode (cat. 6.1248.040, Metrohm, Herisau, Switzerland). An Autolab Potentiostat/Galvanostat (Eco Chemie, The Netherlands) was utilized for electrochemical measurements. Cyclic voltammetry (CV) experiments were performed in 5 mM [Fe(CN)_6_]^3–^/^4–^ containing 0.1 M KCl solution at a scan rate of 50 mV s^–1^. Electrochemical impedance spectroscopy (EIS) was performed at the open-circuit potential (OCP) without a bias voltage in the 0.1–10^4^ Hz frequency range using an alternating current (AC) signal with an amplitude of 10 mV. The electrode surface was modified by the drop-casting method; 6 μL of LNP and **LNPs/CS** solution was drop-cast onto the electrode surface and left to dry at room temperature for 1 h. The experiments were conducted in triplicate.

### HOMO–LUMO Energy Gap

The energy gap between the highest occupied molecular orbital (HOMO) and the lowest-unoccupied molecular orbital (LUMO) of **Np-KLNPs/CS1**, **Np-KLNPs/CS2**, **KLNPs**, and **EHLNPs** was estimated by the Tauc plot according to the equation
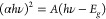
1where *h* is Planck’s constant, ν is the photon frequency, α is the absorption coefficient, *E*_g_ is the band gap, and *A* is a proportionality constant. Exponent 2 indicates indirectly occurring transitions.

## Results and Discussion

### Synthesis and Characterization of Lignin/Chitosan Nanoparticles (LNPs/CS)

**Figure 1:**
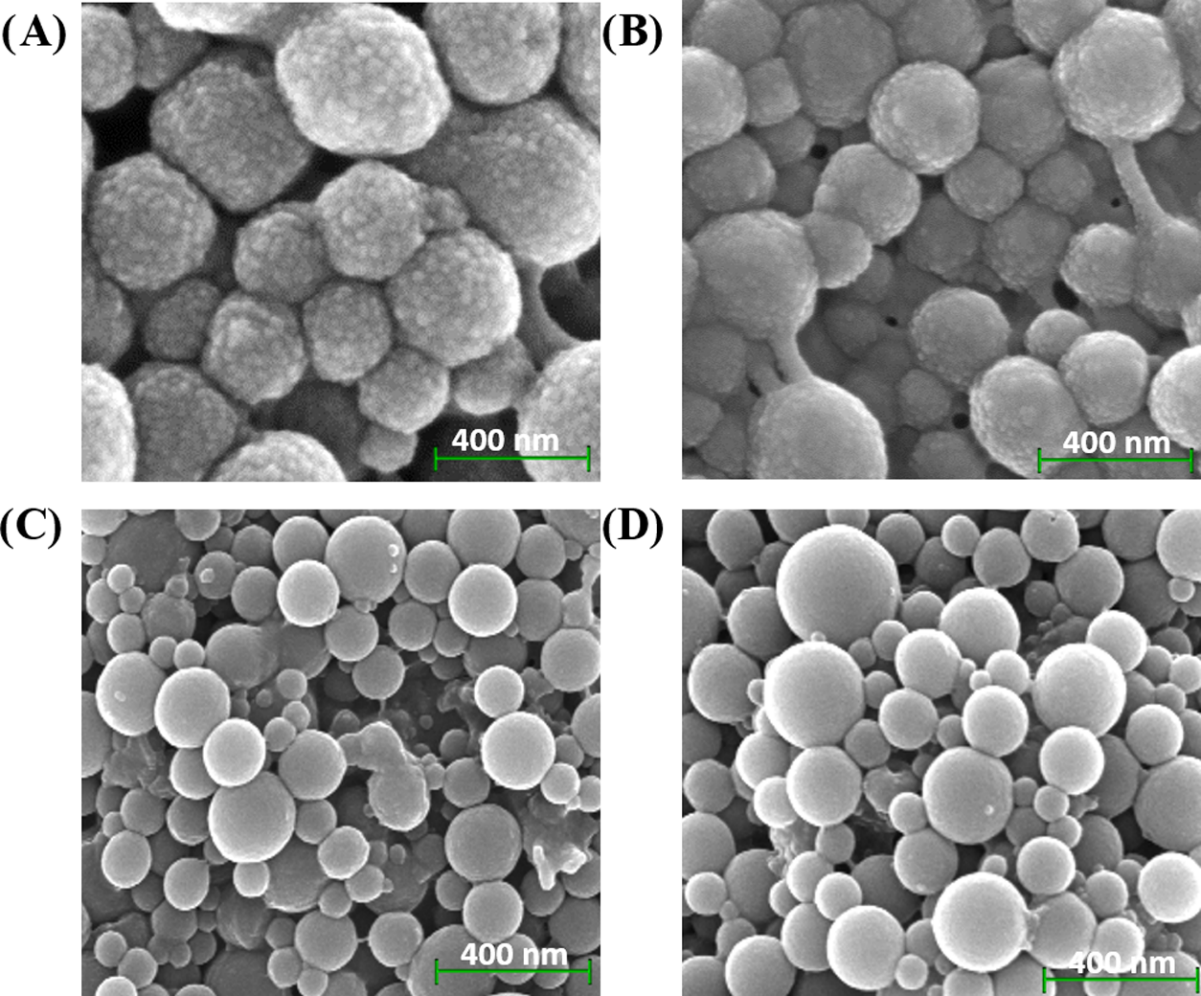
FE-SEM images of **LbL-LNPs/CS** and **Np-LNPs/CS**: (A) **LbL-LNPs/CS**; (B) **Np-LNPs/CS3**; (C) **Np-LNPS/CS1**; and (D) **Np-LNPs/CS2**.

**Scheme 1:**
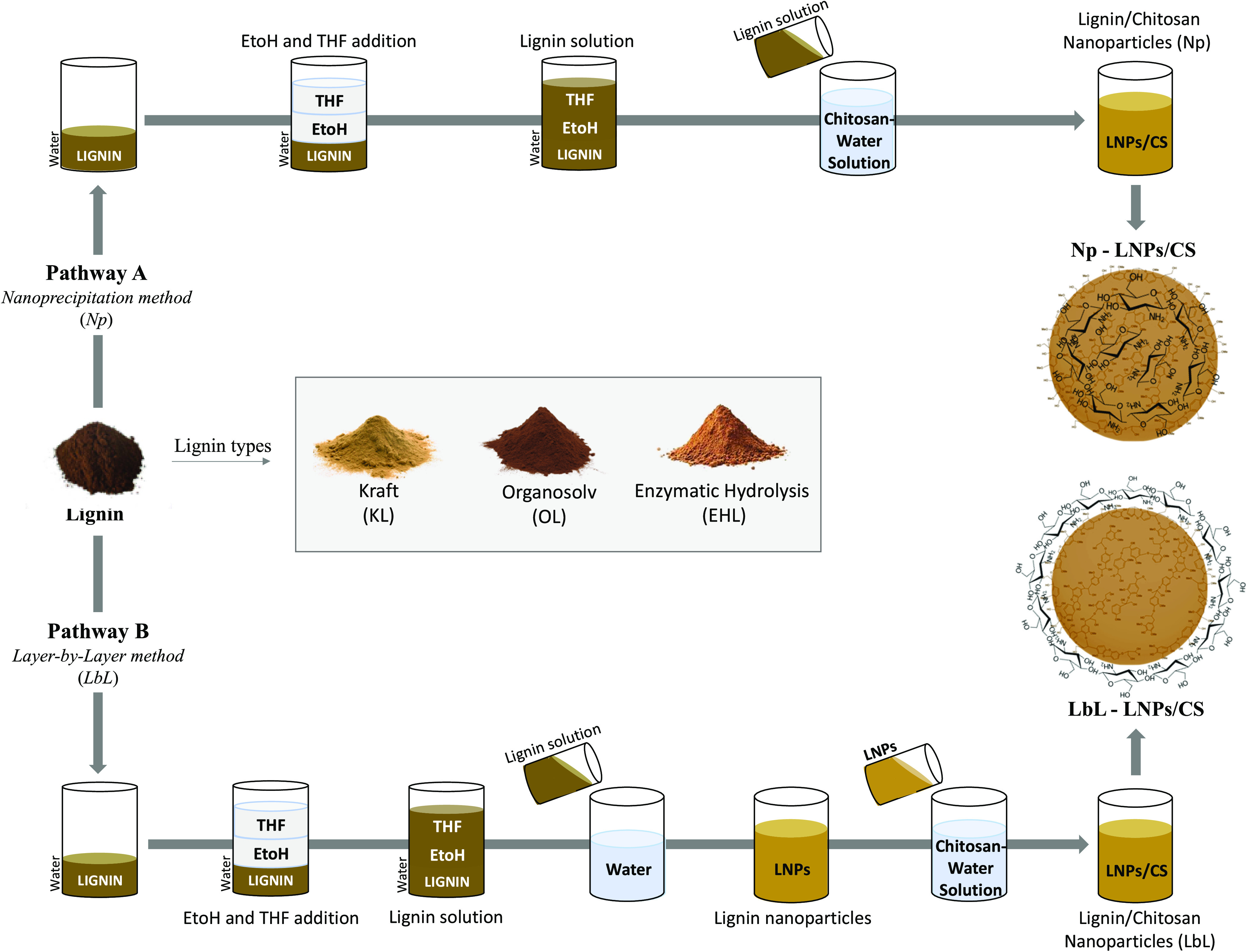
Scheme of Synthesis of **LNPs/CS**: Pathway A: Nanoprecipitation Method; Pathway B: Layer-by-Layer Method

Commercially available KL, OL, and EHL were selected for the preparation of LNPs/CS. KL is a byproduct obtained by the kraft chemical pulping process (c.a. 95% of the overall pulp waste).^44^ OL is derived by the bleaching of lignocellulosic materials with organic solvents at a high temperature, and EHL is a byproduct during the production of biofuel.^45−47^ The distributions of OH functional groups in KL, OL, and EHL were evaluated by quantitative ^31^P NMR analyses and are reported in Table S1. The results were comparable those of KL from softwood, OL switchgrass lignin, and EHL from corncob, respectively.^48−50^ In addition, KL showed the highest amount of guaiacyl OH groups, OL was characterized by the highest amount of aliphatic OH, and EHL by the highest amount of *p*-hydroxyphenyl OH. **LNPs/CS** were prepared by combining KL, OL, and EHL with a sample of chitosan (CS) with different molecular weights (CS1 5 kDa; CS2 50 kDa, and CS3 100 kDa) and a high deacetylation degree (75%). The procedure for the preparation of LNPs/CS is reported in Scheme 1 (pathways A and B). Pathway A involved the nanoprecipitation technology (Np) of the appropriate lignin (1.0 g) dissolved in a ternary mixture of THF/EtOH/Water (14.5 mL) as the primary solvent and CS 1–3 (0.1 g) in deionized water or acetic acid (1.0% water solution) (72.5 mL) as the antisolvent, working under gentle mechanical stirring at 25 °C. **Np-LNPs/CS** were isolated after evaporation of the organic solvent, centrifuged (12,100 rpm for 10 min, 2 times), and freeze-dried.^42,43^ As an alternative, the layer-by-layer (LbL) technology (pathway B) was utilized by coating preformed LNPs (1.0 g) with CS 1–3 (0.1 g) in deionized water, or by acetic acid (1.0% water solution) (72.5 mL), under orbital shaking at 25 °C.^31^**LbL-LNPs/CS** were isolated by centrifugation (12100 rpm for 10 min, 2 times) and freeze-dried. Irrespective of the experimental conditions, the optimal ratio in weight between lignin and CS (1:0.1 ratio) was selected on the basis of previously reported data.^51^ The w/w yield of **Np-LNPs/CS** and **LbL-LNPs/CS** is reported in Table 1. It was always higher than 94% with respect to starting materials. The hydrodynamic diameter values and ζ potentials of **Np-LNPs/CS** and **LbL-LNPs/CS** are presented in Table 1. Dynamic light scattering (DLS) data for original **Np-LNPs/CS** and **LbL-LNPs/CS** are reported in the Supporting Information (Figure S1). **LbL-LNPs/CS** showed an average size (from 352 to 755 nm) higher than that of **Np-LNPs/CS** (from 270 to 710 nm) (Table 1, entries 1–9 *versus* entries 10–18). In addition, they were characterized by a positive ζ potential in the range of +15 to +49 mV, in accordance with the presence of the positively charged chitosan on the surface of LNPs.^52,53^ Conversely, **Np-LNPs/CS** showed a negative ζ potential value in the range of −26 to −53 mV, with the only exception of **Np-LNPs/CS3**, which showed a positive ζ value (Table 1, entries 3, 6, and 9). Thus, chitosan was not significantly deposited on the surface of **Np-LNPs/CS 1–2**. In the latter case, the presence of CS in the core of the nanoparticle structure was highlighted by energy-dispersive X-ray (EDX) spectroscopy analysis of **Np-EHLNPs/CS1** as a selected sample (Figure S2). The field-emission scanning electron microscopy (FE-SEM) analysis of **LbL-LNPs/CS** generally shows the presence of rough aggregates of small clumps on the surface of particles (Figure 1, panel A), representing the structural motif typical of CS.^30,54,55^ A similar behavior was observed in the case of **Np-LNPs/CS3**, confirming that CS was deposited by nanoprecipitation on the surface of the LNPs only in the presence of high-molecular-weight polysaccharides (Figure 1, panel B). In accordance, the small clump-like motif was not visible for **Np-LNPs/CS1** and **Np-LNPs/CS2** (Figure 1, panels C,D, respectively).

**Table 1:** Yield, Size, and ζ-Potential of **Np-LNPs/CS** and **LbL-LNPS-CS**

entry	sample	CS (MW)	yield (%)^a^	size (nm)^b^	ζ-potential
1	Np-KLNPs/CS1	5	96	270	–42
2	Np-KLNPs/CS2	50	96	289	–43
3	Np-KLNPs/CS3	100	96	340	+32
4	Np-OLNPs/CS1	5	96	368	–39
5	Np-OLNPs/CS2	50	95	373	–26
6	Np-OLNPs/CS3	100	94	531	+48
7	Np-EHLNPs/CS1	5	94	672	–53
8	Np-EHLNPs/CS2	50	98	675	–45
9	Np-EHLNPs/CS3	100	97	710	+35
10	LbL-KLNPs/CS1	5	95	352	+15
11	LbL-KLNPs/CS2	50	95	363	+16
12	LbL-KLNPs/CS3	100	95	365	+35
13	LbL-OLNPs/CS1	5	95	545	+24
14	LbL-OLNPs/CS2	50	95	563	+28
15	LbL-OLNPs/CS3	100	94	610	+49
16	LbL-EHLNPs/CS1	5	96	740	+25
17	LbL-EHLNPs/CS2	50	96	742	+28
18	LbL-EHLNPs/CS3	100	94	755	+39

a The yield was calculated as the amount of recovered nanoparticles with respect to the initial amount of lignin and CS.

b The polydispersion index (PDI) of the samples was in the range of 0.20–0.29. The measurements were performed in triplicate at room temperature.

**Figure 2:**
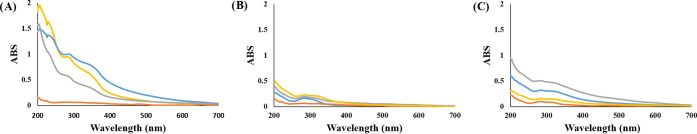
UV–vis absorption spectra of **Np-LNPs/CS** versus **LNPs**: (A) **Np-KLNPs/CS3** (orange line), **Np-KLNPs/CS2** (blue line), **Np-KLNPs/CS1** (yellow line), and **KLNPs** (gray line); (B) **Np-OLNPs/CS3** (orange line), **Np-OLNPs/CS2** (blue line), **Np-OLNPs/CS1** (yellow line), and **OLNPs** (gray line); and (C) **Np-EHLNPs/CS3** (orange line), **Np-EHLNPs/CS2** (blue line), **Np-EHLNPs/CS1** (yellow line), and **EHLNPs** (gray line).

The UV–vis analysis furnished further information on the structural organization of lignin chains in the presence of CS (Figure 2). As a general trend, **LbL-LNPs/CS** absorbed UV radiation with lower efficacy than its Np counterpart, probably due to the presence of the external CS layer (see Figure S3). In the case of **Np-KLNPs/CS1** and **Np-KLNPs/CS2**, the high absorption efficacy was probably related to the optimal π–π interaction between the aromatic subunits of lignin and the low- and medium-molecular-weight CS, as confirmed by the bathochromic effect associated with the ordered head-to-tail structural motif. This hypothesis was in accordance with the contemporary presence of the hyperchromic effect due to the presence of a large number of guaiacyl units.^56,57^ The bathochromic shift was also observed in the case of **Np-OLNPs/CS1** and **Np-OLNPs/CS2**. On the other hand, **Np-KLNPs/CS3** performed differently, probably due to the disorder introduced in the lignin chain aggregation by the high-molecular-weight CS. Finally, **Np-EHLNPs/CS** were the worst absorbing systems, irrespective of the type of CS.

### Electrochemical Characterization of LNPs/CS

**Figure 3:**
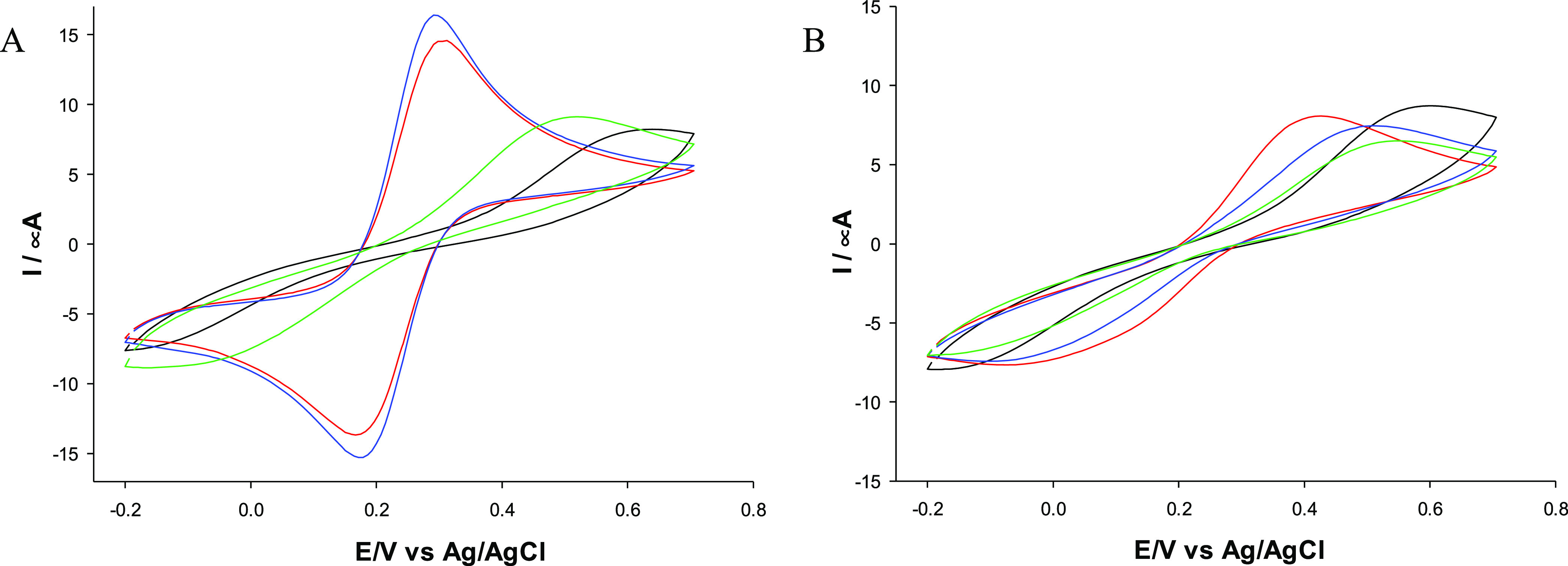
CVs of different KLNP platforms: **KLNPs/GCE** (black line), **KLNPs/CS1/GCE** (red line), **KLNPs/CS2/GCE** (blue line), and **KLNPs/CS3/GCE** (green line). (A) nanoprecipitation method (Np); (B): layer-by-layer method (LbL). Redox probe: 5 mM [Fe(CN)6]^3–/4–^-containing 0.1 M KCl solution. Scan rate: 50 mV s^–1^.

**Figure 4:**
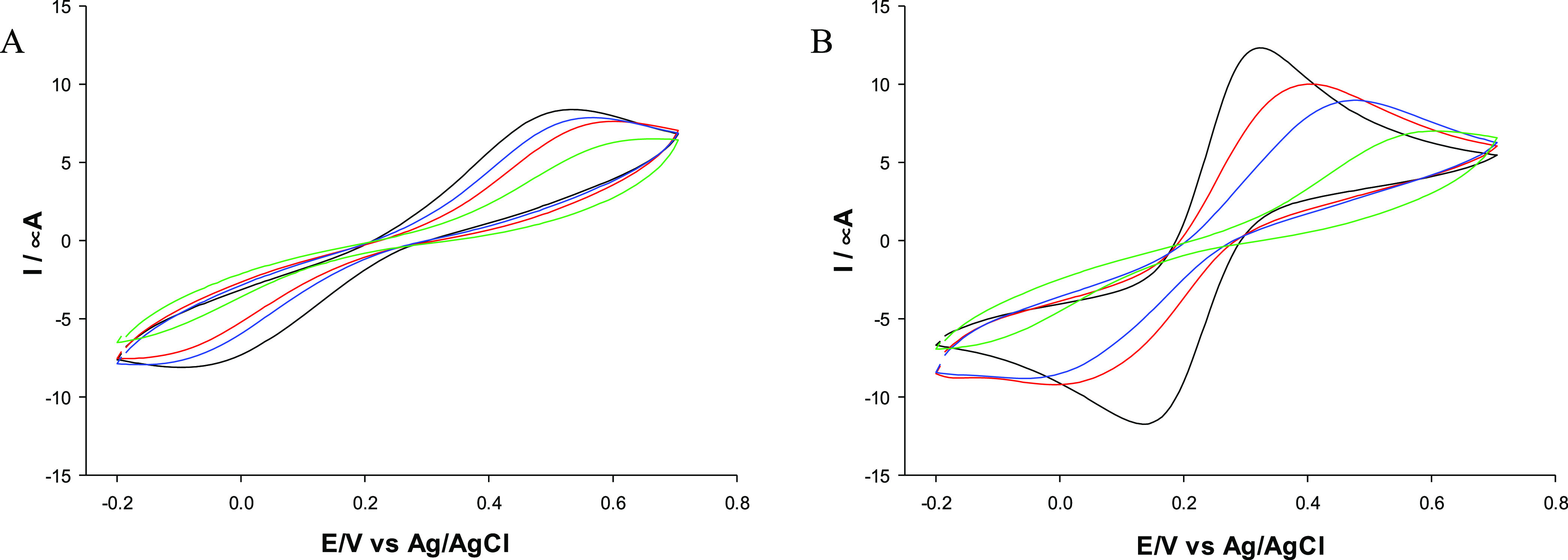
(A) CVs of OLNP platforms by the nanoprecipitation method. **OLNPs/GCE** (black line), **OLNPs/CS1/GCE** (red line), **OLNPs/CS2/GCE** (blue line), and **OLNPs/CS3/GCE** (green line). (B) CVs of EHLNP platforms by the nanoprecipitation method. **EHLNPs/GCE** (black line), **EHLNPs/CS1/GCE** (red line), **EHLNPs/CS2/GCE** (blue line), and **EHLNPs/CS3/GCE** (green line). Redox probe: 5 mM [Fe(CN)6]^3–/4–^-containing 0.1 M KCl solution. Scan rate: 50 mV s^–1^.

LNPs/CS were analyzed by cyclic voltammetry (CV) using a glassy carbon electrode (GCE) modified by the drop-casting technique (**LNPs/CS/GCE)**. CVs were recorded using a solution containing 5 mM [Fe(CN)_6_]^3–/4–^ and 0.1 M KCl at a scan rate of 50 mV s^–1^ (Figures 3 and 4); the electrochemical parameters are presented in Table 2. **Np-KLNPs/CS1/GCE** and **Np-KLNPs/CS2/GCE** showed good electrochemical reversibility (Figure 3, panel A: red and blue lines) in terms of peak current ratios (anodic/cathodic peak current *I*_pa_/*I*_pc_ ≅ 1) and relatively small Δ*E* values of 127 and 113 mV, respectively (Table 2, entries 2 and 3, respectively). In the case of **Np-KLNPs/CS3/GCE** (Figure 3, panel A: green line), an irreversible voltammogram was recorded (Table 2, entry 4). It is important to note that KLNPs showed a totally irreversible electrochemical behavior (Figure 3, panel A: black line), with a very large potential peak separation (Δ*E*) corresponding to 570 mV (Table 2, entry 1). Thus, the presence of CS improved the electrochemical responsiveness of KLNPs during the nanoprecipitation procedure. The **LbL-KLNPs/CS/GCE** platforms showed irreversible voltammograms (Figure 3, panel B), irrespective of the type of CS used as a layer, confirming a very low electron-transfer rate (Table 2, entries 5–7). On the basis of these data, the Np technology served as the most efficient method for the preparation of electroactive lignin nanodevices. In principle, the layer-by-layer method produced well-defined aggregates in which lignin nanoparticles are in the core and chitosan is on the particle surface. Chitosan, present as an external layer, acts as an insulating agent, limiting the electrochemical responsiveness of the overall platform. Conversely, the nanoprecipitation method produces random aggregates between lignin and chitosan. These random interactions lead to productive or unproductive electrochemical platforms. In addition, low- and medium-molecular-weight CS were more effective than their high-molecular-weight counterpart in improving the electrochemical behavior of the nanomodified platform. A similar trend was reported in our previous study in correlation to the steric hindrance of chitosan, with finely tuned antioxidant and UV-absorbing properties of nanoparticles of lignin and saccharides from fishery waste.^30^

**Table 2:** Comparison of Electrochemical and Electronic Parameters from Tauc Plot Calculations

entry	platform	*I*_pa_ (μA)	*I*_pc_ (μA)	Δ*E* (mV)
1	KLNPs	2.76	–1.74	570 (3.8)
2	Np-KLNPs/CS1	14.88	–13.11	127 (3.3)^a^
3	Np-KLNPs/CS2	16.91	–14.84	113 (3.1)^a^
4	Np-KLNPs/CS3	5.74	–3.77	470
5	LbL-KLNPs/CS1	6.49	–3.22	344
6	LbL-KLNPs/CS2	4.38	–2.51	463
7	LbL-KLNPs/CS3	3.01	–1.05	494
8	OLNPs	5.89	–4.36	407
9	Np-OLNPs/CS1	3.43	–1.24	589
10	Np-OLNPs/CS2	4.22	–1.60	546
11	Np-OLNPs/CS3	2.23	–0.81	666
12	LbL-OLNPs/CS1	4.12	–2.98	449
13	LbL-OLNPs/CS2	5.23	–3.01	421
14	LbL-OLNPs/CS3	2.17	–0.92	648
15	EHLNPs	13.54	–11.56	169 (3.1)^a^
16	Np-EHLNPs/CS1	8.31	–6.13	281
17	Np-EHLNPs/CS2	5.12	–3.56	435
18	Np-EHLNPs/CS3	2.01	–1.04	653
19	LbL-EHLNPs/CS1	5.66	–3.54	451
20	LbL-EHLNPs/CS2	8.01	–5.94	301
21	LbL-EHLNPs/CS3	4.16	–2.01	516

a *E*_g_ (eV).

It is interesting to note that the Δ*E* values of **Np-KLNPs/CS1/GCE** and **Np-KLNPs/CS2/GCE** are of the same order of magnitude as those of previously reported electrochemical platforms based on carbon black (Δ*E* values in the range of 120–180 mV) and gold nanoparticles (Δ*E* values of 100 mV).^58,59^**Np-OLNPs/CS/GCE** and **Np-EHLNPs/CS/GCE** (Figure 4, panels A and B, respectively) showed no electrochemical reversibility (Table 2, entries 9–11 and 16–18, respectively). Again, no significant electrochemical reversibility was observed for **LbL-OLNPs/CS/GCE** and **LbL-EHLNPs/CS/GCE** (Figure S4 and Table 2 entries 12–14 and 19–21, respectively). As EHLNPs alone showed good electrochemical reversibility with a Δ*E* value of 169 mV (Figure 4, panel B, black line; Table 2, entry 15), the loss of electrochemical efficacy shown by **EHLNPs/CS/GCE** can be ascribed to the steric effect exerted by the presence of CS.

The values of the real electroactive area (*A*_EA_) of the platforms, which showed the best results in terms of electrochemical responsiveness, were determined by calculating the slope of the *i*_p_ vs *v*^1/2^ plot and successively inserting this value into the following Randles–Sevcik equation
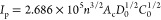
2where *I*_p_ is the voltammetric peak current (*A*), *n* is the number of electrons, *A*_EA_ is the electroactive area (cm^2^), *D*_0_ is the diffusion coefficient (7.6 × 10^–6^ cm^2^ s^–1^ for ferricyanide), *C*_0_ is the concentration (mol cm^–3^), and *v* is the scan rate (V s^–1^). The real *A*_EA_ values resulted in the following order: 0.31 cm^2^ for Np-KLNPs/CS-2/GCE > 0.24 cm^2^ for Np-KLNPs/CS-1/GCE > 0.19 cm^2^ for EHLNPs/GCE. A marked increase of *A*_EA_ for all platforms was observed compared to the value of the geometrical area (0.125 cm^2^), confirming the high capability of nanoparticles to increase the electrode surface area and, therefore, enhance the electrochemical performances of the nanomodified electrodes.

The stability of all platforms was also evaluated by repeating the CVs after 10 min. No significant difference was observed in all voltammograms recorded (curves not shown).

**Figure 5:**
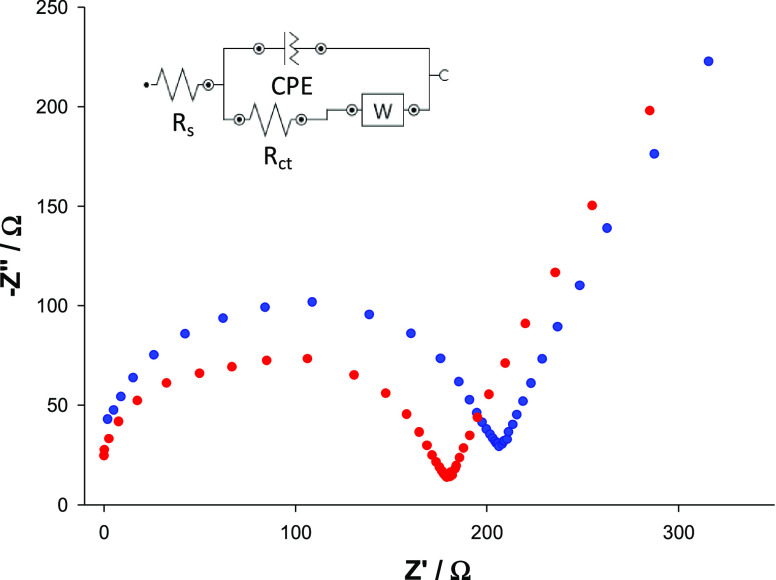
Nyquist plots of **Np-KLNPs/CS2/GCE** (red curve) and **Np-OLNPs/CS3/GCE** (blue curve), measured in 5 mM [Fe(CN)_6_]^3–/4–^-containing 0.1 M KCl solution. The inset shows the equivalent circuit used for fitting the experimental data.

Next, systems with the best and the worst electrochemical responsiveness (**Np-KLNPs/CS2/GCE** and **Np-OLNPs/CS3/GCE**) were characterized by electrochemical impedance spectroscopy (EIS) technique. Charge-transfer resistance (*R*_ct_) was evaluated by the Nyquist plots and recorded in 5 mM [Fe(CN)_6_]^3–/4–^ solution (Figure 5, red and blue curves, respectively). The impedance spectra were fitted by using the Randles circuit [R(Q[RW])]. The *R*_ct_ value of **Np-KLNPs/CS2/GCE (**181 Ω) was lower than that of **Np-OLNPs/CS2/GCE** (217 Ω), further confirming the beneficial electrochemical properties of the aggregate between KL and CS2.

### Determination of the HOMO–LUMO Gap

**Figure 6:**
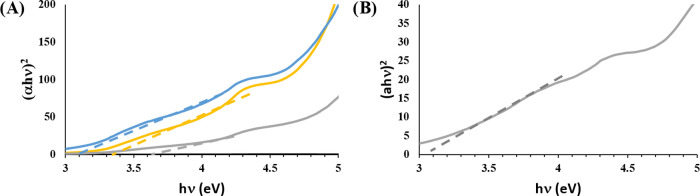
Tauc’s plot of **Np-KLNPs/CS1**, **Np-KLNPs/CS2**, and EHLNPs. (A) **Np-KLNPs/CS1** (yellow line), **Np-KLNPs/CS2** (blue line), and KLNPs (gray line); (B) EHLNPs (gray line).

The HOMO–LUMO energy gap was applied to rationalize the electrochemical behavior of LNPs/CS.^60^ The Tauc plots of Np-LNPS/CS samples are depicted in Figure 6, while the optical band gap of the sample is shown in Table 2. We were able to calculate the HOMO–LUMO energy gap only in the cases of **Np-KLNPs/CS1**, **Np-KLNPs/CS2**, KLNPs, and EHLNPs. The most electrochemically responsive systems (**Np-KLNPs/CS1**, **Np-KLNPs/CS2**, and **EHLNPs**) showed a lower value of the HOMO–LUMO energy gap with respect to the parent lignin (Table 2, entries 2, 3, and 15). As a low value of the HOMO–LUMO energy gap reflects the intensity of π–π interactions in head-to-tail-oriented aromatic subunits, the highest electrochemical reversibility was associated with nanostructures possessing the highest internal order.^61^ In this scenario, CS played the role of a structural probe in orienting the lignin chains from an unfavorable to a favorable head-to-tail configuration (on/off switch). This effect was particularly relevant in the case of KL, where the electrochemical responsiveness was effective only in the presence of CS. In contrast, CS inhibited the original electrochemical responsiveness of EHL, exerting an unfavorable steric effect.

## Conclusions

Composite nanoparticles prepared from lignin and chitosan by layer-by-layer technology and nanoprecipitation procedure were studied for their electrochemical responsiveness. The electrochemical reversibility of these nanoparticles was found to be strictly dependent on the preparation procedure, the molecular weight of chitosan, and the type of lignin. As a general trend, the nanoprecipitation procedure associated with the use of KL afforded the most electrochemically responsive nanodevice, irrespective of the chitosan type, suggesting that the aggregation during the nanoprecipitation process promotes the most effective electrochemical interactions between the two biopolymers. The relevance of the interaction between chitosan and lignin in the electrochemical responsiveness of a nanodevice was further highlighted by the effect of the steric hindrance of chitosan. Low- and medium-molecular-weight chitosan yielded nanoparticles with the highest electrochemical reversibility (Δ*E* value in the range of 127–113 mV). As shown by FE-SEM and X-ray analyses, this type of chitosan was embedded in the core of nanoparticles, favoring the formation of head-to-tail aggregates characterized by low values of the HOMO–LUMO energy gap. In contrast, high-molecular-weight chitosan significantly modified the structure of the aggregate, also emerging from the surface of nanoparticles as a small clump-like motif. In addition, the beneficial effect of chitosan is synergic with the type of lignin partner. While a positive effect was observed for KL (and in minor amounts for OL), it became detrimental in the case of EHL. This result was probably due to the higher amount of *p*-hydroxyphenyl subunits present in EHL than in KL and OL, which can give rise to different CS interaction networks with respect to guaiacyl subunits.^62,63^ The easy preparation of LNPs/CS by the nanoprecipitation process, associated with a greater sustainability and electrochemical efficiency of these nanodevices in comparison to commercial references, suggests that LNPs/CS is a sustainable alternative for the design of new eco-sustainable electrochemical platforms.

## Supporting Information Available

Table S1: functional group distribution as derived by quantitative ^31^P NMR analyses of the samples; Figure S1**:** dynamic light scattering (DLS) data for **Np-LNPs/CS** and **LbL-LNPs/CS**; Figure S2**:** energy-dispersive X-ray (EDX) spectra of **Np-EHLNPs/CS1**; Figure S3: UV–vis absorption spectra of **LbL-LNPs**; Figure S4: CVs of **LbL-OLNPs/CS/GCE** and **LbL-EHLNPs/CS/GCE** (PDF)
