# Supplementary material for: Tuning the Effect of Chitosan on the Electrochemical Responsiveness of Lignin Nanoparticles
Source: ACS Biomater Sci Eng. 2023 May 16;9(6):3597–605. doi: 10.1021/acsbiomaterials.2c01494 (PMC10265579; doi:10.1021/acsbiomaterials.2c01494)
Supplement: Supplementary file 1 — ab2c01494_si_001.pdf [file ab2c01494_si_001.pdf]

Supporting Information

Tuning Effect of Chitosan in Electrochemical Responsiveness of Lignin Nanoparticles

*Valeria Gigli[1], Eliana Capecchi[2, *], Cristina Tortolini[1], Andrea Isidori[1], Riccarda Antiochia[3, *], Raffaele Saladino[2]*

[1] Department of Experimental Medicine, Sapienza University of Rome, Viale Regina Elena 324, 00166 Rome, Italy.

[2] Department of Biological and Ecological Sciences, University of Tuscia, 01100 Viterbo, Italy.

[3] Department of Chemistry and Drug Technologies, Sapienza University of Rome, Piazzale Aldo Moro 5, 00185 Rome, Italy.

[*] Correspondence: riccarda.antiochia@uniroma1.it; e.capecchi@unitus.it.

Table S1. Functional group distribution as derived by quantitative ^{31}P NMR analyses of the samples.

**Lignin sample**	**EHL**	**OL**	**KL**
**Lignin Hydroxyl Group[a]**	**Abundance (mmol/g)[a]**		
Aliphatic OH	1.639	3.367	1.891
C_5 substituted/condensed OH	1.466	0.533	0.270
guaiacyl OH	1.615	0.670	3.853

p-hydroxyphenyl OH	1.108	0.420	0.286
carboxylic acid OH	0.966	0.317	0.453

[a] Error for NMR quantification data was estimated to be ±0.1 mmol/g

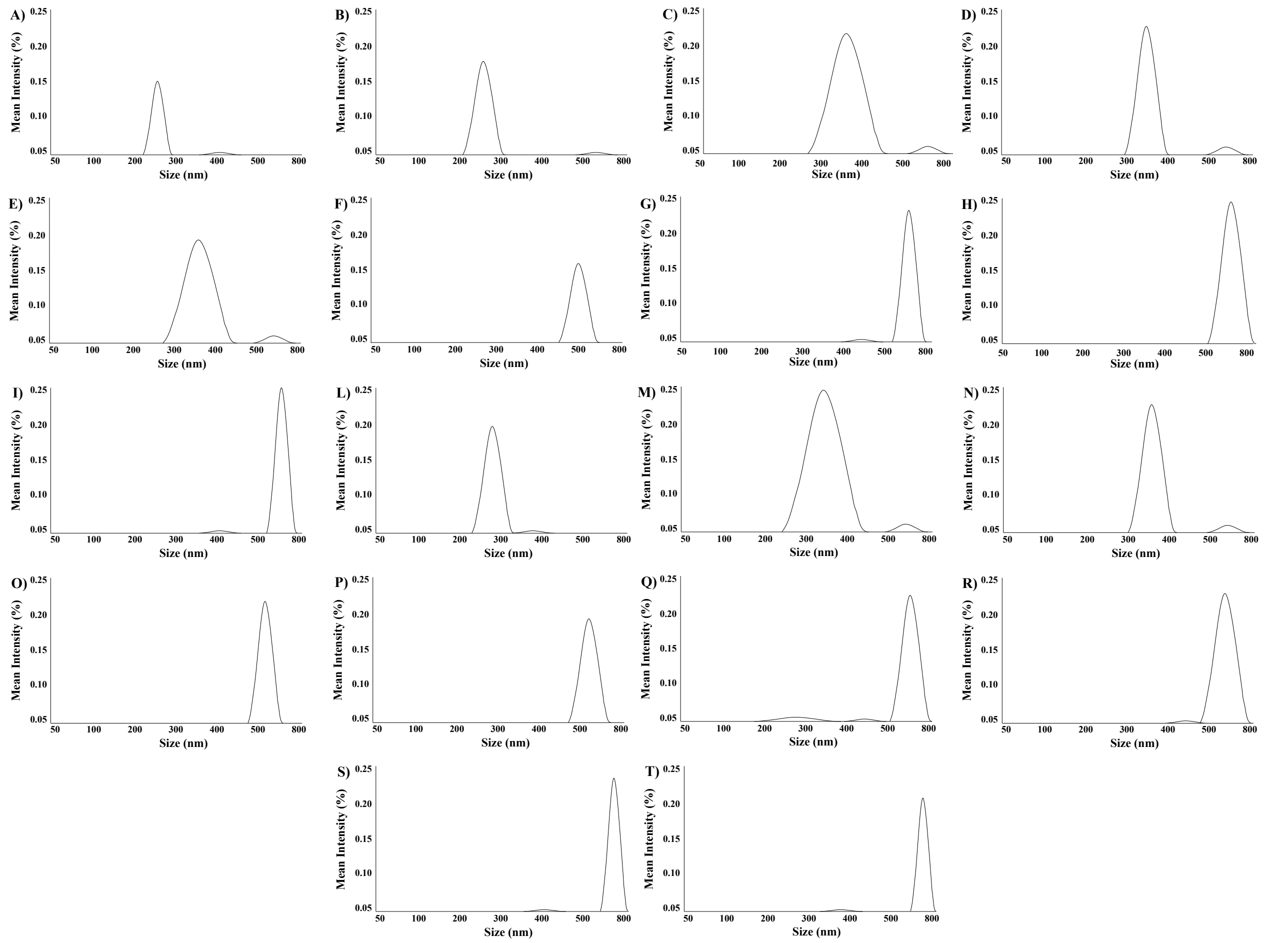

Figure S1. Dynamic Light Scattering (DLS) of Np-LNPs/CS and LbL-LNPs/CS. Panel A: Np-KLNPs/CS1. Panel B: Np-KLNPs/CS2. Panel C: Np-KLNPs/CS3. Panel D: Np-OLNPs/CS1. Panel E: Np-OLNPs/CS2. Panel F: Np-OLNPs/CS3. Panel G: Np-EHLNPs/CS1. Panel H: Np-EHLNPs/CS2. Panel I: Np-EHLNPs/CS3. Panel L: LbL-KLNPs/CS1. Panel M: LbL-KLNPs/CS2. Panel N: LbL-KLNPs/CS3. Panel O: LbL-OLNPs/CS1. Panel P: LbL-OLNPs/CS2. Panel Q: LbL-OLNPs/CS3. Panel R: LbL-EHLNPs/CS1. Panel S: LbL-EHLNPs/CS2. Panel T: LbL-EHLNPs/CS3.

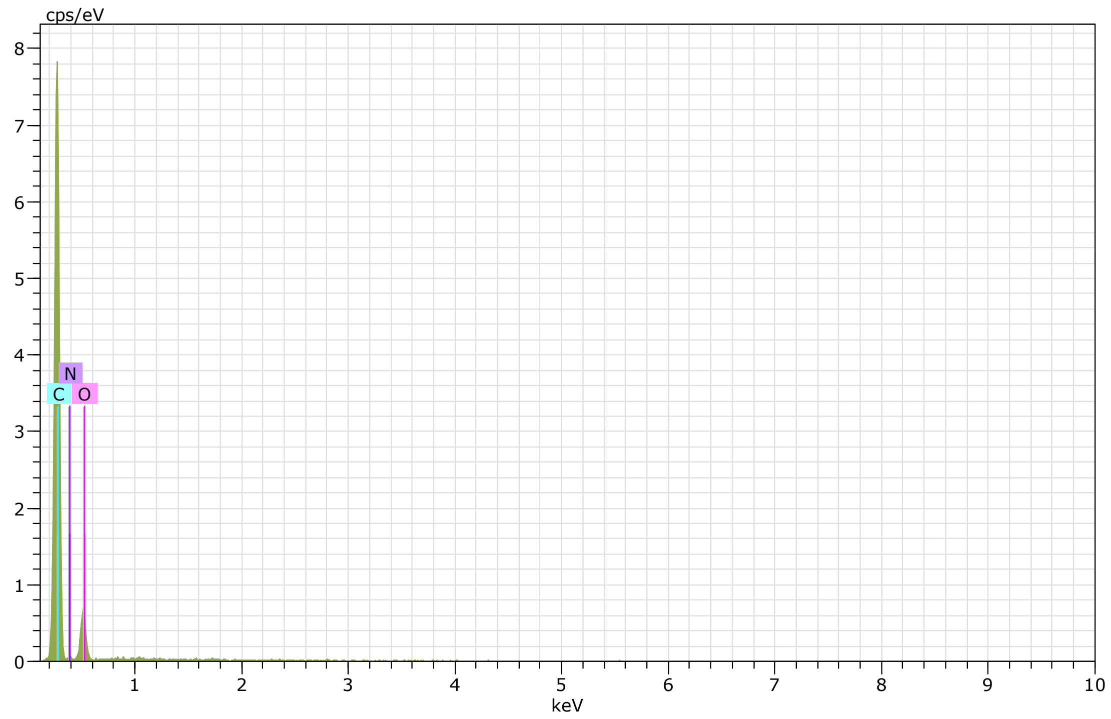

Figure S2. Energy-dispersive x-ray spectrometry (EDX) spectra of **Np-EHLNPs/CS1**.

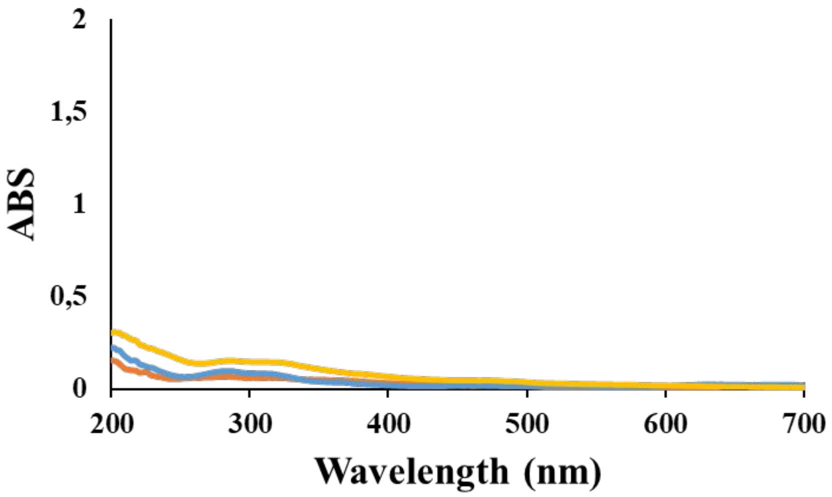

Figure S3. UV-vis absorption spectra of **LbL-LNPs.**

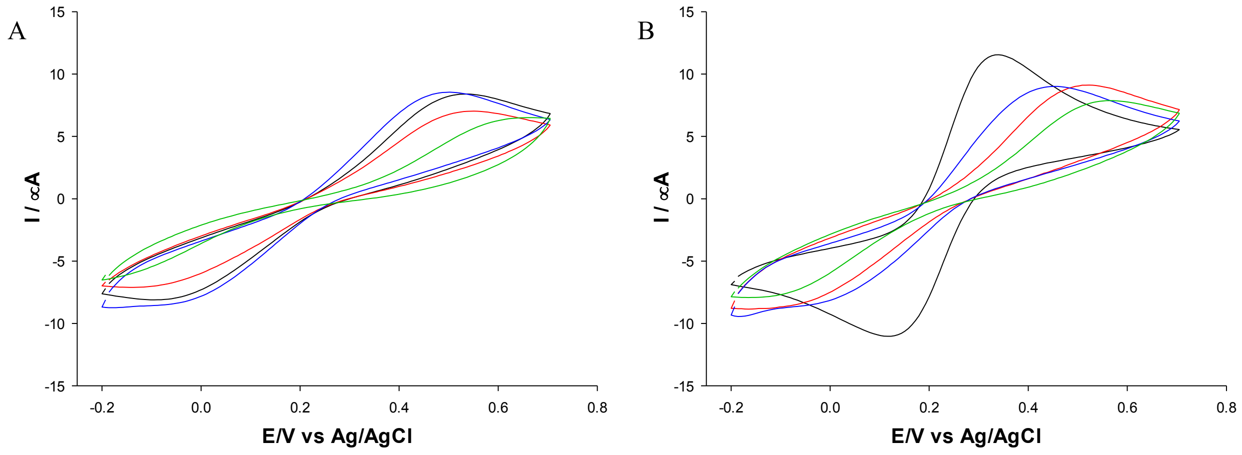

Figure S4. CVs of LbL-OLNPs/CS/GCE and LbL-EHLNPs/CS/GCE. Panel A: OLNPs/GCE (black line), LbL-OLNPs/CS1/GCE (red line), LbL-OLNPs/CS2/GCE (blue line) and LbL-OLNPs/CS3/GCE (green line). Panel B: EHLNPs/GCE (black line), LbL-EHLNPs/CS1/GCE (red line), LbL-EHLNPs/CS2/GCE (blue line) and LbL-EHLNPs/CS3/GCE (green line). Redox probe: 5mM $[Fe(CN)_6]^{3-/4-}$containing 0.1 M KCl solution. Scan rate: 50 mV s^{-1}.
